# A nomogram-based immunoprofile predicts overall survival for previously untreated patients with esophageal squamous cell carcinoma after esophagectomy

**DOI:** 10.1186/s40425-018-0418-7

**Published:** 2018-10-03

**Authors:** Jingjing Duan, Yongwei Xie, Lijuan Qu, Lingxiong Wang, Shunkai Zhou, Yu Wang, Zhongyi Fan, Shengsheng Yang, Shunchang Jiao

**Affiliations:** 1 School of Medicine, Nankai University, Tianjin, 300071 China; 2 Department of Oncology, Oncology Laboratory, General Hospital of Chinese PLA, Beijing, 100853 China; 3 Department of Cardio-Thoracic Surgery, Fuzhou General Hospital, Fuzhou, 350025 China; 4 Department of Pathology, Fuzhou General Hospital, Fuzhou, 350025 China

**Keywords:** Esophageal squamous cell carcinoma, Nomogram, Immunoprofile, CD8, PD-L1

## Abstract

**Background:**

Immunoscore, as a prognostic tool defined to quantify in situ immune cell infiltrates, appears to be superior to the TNM staging system. In esophageal squamous cell carcinoma (ESCC), no immunoscore has been established; however, in situ tumor immunology is recognized as highly important. Our study aimed to construct a comprehensive immunoprofile for ESCC.

**Methods:**

The infiltration of four immune cell types (CD8+/CD4+/Foxp3+/CD33+ cells), the expression of both inhibitory (PD-1/PD-L1/Tim-3/LAG-3) and stimulatory checkpoints (OX-40/ICOS), and IDO1 were evaluated by IHC staining and multi-color immunofluorescence in two independent cohorts (95 patients in the primary cohort and 55 patients in the validation cohort). The association with patients’ overall survival was analyzed by the Kaplan-Meier method and the Cox model. Nomogram-based immunoprofile was established using the independent prognostic variables. To determine its predictive accuracy and discriminatory capacity, the C-index and calibration curve were calculated.

**Results:**

Significant correlation of PD-L1 expression in tumor cells with PD-1+ T cell infiltration was found (*P* = 0.035), indicating the activation of the inhibitory PD-1/PD-L1 pathway in ESCC cases. More PD-L1+ ICs, Tim-3+ ICs and LAG-3+ ICs were found in the CD8-rich tumor microenvironment, which is in accordance with the feedback nature of immune system. After adjustment by TNM stage, four immune variables including the infiltration of CD8+/Foxp3+/CD33+ cells and the PD-L1 expression by tumor cells were selected to construct a prognostic nomogram. The calibration curves showed good accuracy of the nomogram for survival prediction. To overcome the complexity of applying a nomogram in a clinical setting, a simple immunoprofile was then established according to the points of each factor from the nomogram. Our immunoprofile model could separate same-stage patients into different risk subgroups, and showed superior accuracy for survival prediction than the TNM staging system based on the C-index calculation and ROC analysis.

**Conclusions:**

Our nomogram-based immunoprofile can provide more accurate prognosis prediction and is an important complement to the TNM staging system for operable ESCC patients.

**Electronic supplementary material:**

The online version of this article (10.1186/s40425-018-0418-7) contains supplementary material, which is available to authorized users.

## Background

Although esophageal adenocarcinoma is predominant in the western countries, esophageal squamous cell carcinoma (ESCC) accounts for the bulk of cancer incidence and mortality in China [1, 2]. Esophagectomy is by far the priority and the best curative option for patients with local or locoregional ESCC in China. As with the other cancers, prognostic information for ESCC after surgery is extremely important. The most widely used staging system for ESCC is the TNM classification system. However, the clinical outcome may be significantly different in the patients with the same TNM stage. Therefore, researchers are intensively looking for additional factors that are highly related to the prognosis. A large number of studies have revealed that immune factors in the tumor microenvironment (TME) have a significant impact on the prognosis of cancer patients [3–7]. At present, a TNM-immune system, which is used to evaluate the prognosis of colorectal cancer patients, has been preliminarily constructed [8, 9]. The evaluation system includes the infiltration density and location of CD3/CD8+ T cells and is proved to be superior to the TNM staging system in judging the prognosis [10]. However, at this time, a prognostic immunoassay system for ESCC has not been identified.

The tumor immune microenvironment, including a variety of immune cells and immunomodulatory molecules, plays an important role in inhibiting or enhancing the anti-tumor immune response [11, 12]. There are many immune effector cells such as CD8+ T cells and CD4+ T cells, and various immunosuppressive cells such as regulatory T (Treg) cells and myeloid derived suppressor cells (MDSCs) infiltrating in the tumor site. However, the correlation between the infiltration of defined immune cells to clinical outcome in different cancers is controversial. For example, high density of Treg cells predicted worse survival in breast cancer [13] whereas favorable survival in colon cancer [14]. The prognostic role of different immune cell infiltration in ESCC is still unclear.

Immune escape is a hallmark of tumor progression. PD-1/PD-L1 pathway is one of the most important signaling pathways that mediate tumor immune escape. PD-1/PD-L1 blockade can induce a more durable response in patients with advanced cancer [15–17]. The results of KEYNOTE-028 showed that the overall response rate (ORR) of Pembrolizumab treatment could reach 30% in advanced esophageal cancer patients who had PD-L1-positive tumors, and the ORR was 29% in squamous cell type [18]. Another clinical trial showed that the ORR of Nivolumab treatment could reach 17% in patients with treatment-refractory ESCC [19]. Thus, blocking of PD-L1/PD-1 is effective in the treatment of ESCC. However, the prognostic value of PD-L1 remains controversial [5, 20, 21]. PD-L1 can be expressed by a variety of cell types including tumor cells and stromal cells; whether PD-L1 expressed by different cells has different prognostic value is unknown. In addition to the PD-1/PD-L1 pathway, the function of other immune checkpoints such as Tim-3, LAG-3, OX-40, ICOS has also been explored [22–24] and some inhibitors have been investigated in clinical trials. The prognostic significance of these immune checkpoints in ESCC remains unknown. Furthermore, indolamine − 2,3- dioxygenase 1 (IDO1) catalyzing tryptophan to kynurenine is another important target. Tumor cells achieve immune tolerance with the help of IDO1. The combination of PD-1 inhibitors and IDO1 inhibitors are promising [25, 26] and under-investigated. The prognostic value of IDO1 in ESCC needs to be further confirmed.

The anti-tumor immune response is complicated, a more reliable model incorporating multiple immune effectors is necessary to help judge the prognosis of cancer patients. Nomogram, a statistical predictive model, by scoring every prognostic variable for each individual patient, has shown better accuracy for survival prediction than TNM staging. As a visual representation of the hazard ratios of multiple variables, nomogram can provide more accurate clues to quantify each factor in the final immunoprofile. In current study, the infiltration of four immune cell types (CD8+ /CD4+/Foxp3+/CD33+ cells), the expression of both inhibitory (PD-1/PD-L1/Tim-3/LAG-3) and stimulatory checkpoints (OX-40/ICOS), and IDO1 were evaluated in two independent cohorts with ESCC (95 patients in the primary cohort and 55 patients in the validation cohort). A nomogram-based immunoprofile comprised of independent prognostic variables was then constructed and validated. This approach can further divide patients with the same TNM staging into subgroups with different risk and shows a superior capacity in survival prediction than TNM staging.

## Methods

### Study design and patients

This study was conducted on two independent cohorts of ESCC patients from two hospitals. The inclusion criteria were as follows: no history of other malignancies; no neoadjuvant therapy; no unresectable tumors or distant metastases; histopathologically verified ESCC; with complete clinicopathological and follow-up information; and received standardized combination chemotherapy if relapsed. The exclusion criteria included the following: perioperative mortality and developed a second primary cancer during follow-up. The primary cohort included 95 previously untreated patients who underwent esophagectomy in the Fuzhou General Hospital between May 2010 and June 2011. Patients from the General Hospital of Chinese PLA at the same period were assigned to the validation cohort (*n* = 55).

All patients were categorized according to the AJCC 8th TNM staging system. The median follow-up period was 32 months (range, 3–84 months) in the primary cohort and 33 months (range, 3–98 months) in the validation cohort. Overall survival (OS) was defined as the period from the date of esophagectomy to the date of death, or until the last follow-up visit. Those who were still alive in June 2018 were considered as censored data. The study was approved by the institutional ethics committee and all procedures were conducted in accordance with ethical principles.

### Immunohistochemistry (IHC) and scoring

IHC assays for CD8, CD4, FOXP3, CD33, PD-1, PD-L1, Tim-3, LAG3, OX-40, ICOS and IDO1 were performed on primary surgical specimen using standard indirect immunoperoxidase protocols. Briefly, embedded tumor tissues were sectioned to 3-μm thickness, deparaffinized twice with xylene and rehydrated in a graded series of ethanol. Heat-mediated antigen retrieval in citrate buffer was performed followed by 3% hydrogen peroxide. After blocked with serum, sections were incubated with indicated primary antibodies at appropriate dilution at 4 °C overnight. The information of primary antibodies used in IHC was listed in Additional file 1. The next day, the sections were incubated with second antibodies for 30 min at room temperature and visualized by staining with the DAB system, then counterstained with hematoxylin, dehydrated, and coverslipped.

For the evaluation of IHC assays, all specimens were examined independently by two experienced pathologists in a blinded manner. Semiquantitative analyses of TILs were performed on full slides, and the results were estimated as relative percentage staining. The staining was firstly evaluated according to overall impression at low microscopic magnification (100X) and calculated as the mean value of five random field at higher magnification (200X). Staining of CD8/CD4 cells was evaluated both in tumor parenchyma and mesenchyme area, and was defined as CD8-poor if infiltration < 1% in parenchyma and < 10% in mesenchyme at the same time, CD8-rich for others; CD4-poor if infiltration < 1% in parenchyma and < 8% in mesenchyme at the same time, CD4-rich for others. Staining pattern of Foxp3+/CD33+ cells was considered as negative or positive if <1% or ≥ 1%, respectively. PD-L1 was expressed both on the surface of tumor cells (TCs) and immune cells (ICs), and was regarded as negative or positive if <1% or ≥ 1% in TCs, respectively, or if <1% or ≥ 1% in ICs, respectively. For the remaining checkpoints, the expression pattern was defined as positive if ≥1% in ICs. Staining of IDO1 was also evaluating separately in TCs and in ICs, and was considered as positive if staining area ≥ 1%. For the subsequent statistical analyses, each biomarker was recorded as a dichotomous (high vs. low) variable by the optimal cut-off value using the minimum *P* value approach.

### Multi-color immunofluorescence (IF) and automatic counting

Multi-color IF for CD8+ TIL, Foxp3+ TIL, CD33+ MDSC and CK expression in tissue sections was performed using OPAL-5-color reagents (Perkin-Elmer) according to the manufacturer’s instructions. Briefly, tissue blocks were cut into 3-μm slices, and dewaxed and rehydrated as the IHC assay. The sections were performed antigen retrieval with citrate buffer in microwave (the retrieval buffer was first brought to boiling point at 100% power and then an additional 15 min at 20% power). After blocked with serum for 30 min, the slides were incubated with the first primary antibody for 2 h at room temperature. Sections were further incubated with according secondary antibody for anther 30 min at room temperature. After washed thrice in TBST, the tissue sections were incubated with the Opal Working Solution to generate the Opal signal (10 min at room temperature). The microwave treatment was then performed followed by the second marker staining. After the last microwave treatment, the slides were stained with DAPI and then coverslipped. The information of primary antibodies used in IF was listed in Additional file 1.

Five random images from each section at high magnification (200X) were acquired on the Vectra Automated Quantitative Pathology Imaging System (Perkin-Elmer). The positive cells in each image were automatically counted using the Inform software (Perkin-Elmer) and were recorded as the mean value. For the quantitative analysis of multi-color IF, although no training was done between the pathologists and the inform software, a series optimal experimental processes and analysis procedures have been carried out to reduce the deviation. First of all, the dyeing and analysis of each single marker were performed and then adjusted to ensure that the exposure intensity of each biomarker was consistent in the multicolor experiment, which is conducive to the subsequent analysis of fluorescent signals. Secondly, two thresholds were used in the analysis processes in order to guarantee the comparability of results on different slides. The one is the minimum region signal threshold used to identify the true positive region; the other is the cell positivity threshold selected for the exclusion of nonspecific or false positive cells. Based on the standard set of rules to identify the true positive cells, the interpretation results of fluorescent signals are credible and comparable.

### Statistical analyses

All statistical analyses were performed using IBM SPSS Statistics, Version 20.0. Characteristics of the patients were described with percentages or median values. Categorical variables were compared using the χ^2^ test or Fisher’s exact test. Continuous variables were managed using the t test. When the variables were ordinal, non-parametric test was conducted. The correlation between the IHC scoring and the results of IF counting was estimated by the coefficient of Person. Survival curves were estimated with the Kaplan-Meier method and compared using the log-rank test. The univariate and multivariate Cox analyses were performed to determine the independent risk characteristics. Hazard ratios (HRs) and 95% confidence intervals (CIs) of these variables were estimated to quantify the strength of these associations. All statistical tests were 2-tailed. A *P* value of < 0.05 was considered as statistically significant.

### Development of the prognostic nomogram and immunoprofile system

A nomogram that can visualize the prognostic strength of different risk factors in a single figure was established using the package of rms in R, version 3.4.2(http://www.r-project.org/). The factors used to construct the prognostic nomogram were selected based on the Cox proportional hazards regression model using the backward stepwise selection with the Akaike information criterion. The internal validation of the nomogram was conducted by bootstraps with 1000 resamples. The external validation was then performed using the validation cohort. The concordance index (C-index) and calibration curve were used to determine its predictive accuracy and discriminatory capacity. The C-index of the TNM staging system was calculated. The larger C-index, the more favorable predictive accuracy of the model.

To simply the nomogram, we then established an immunoprofile based on the points in the nomogram. The prognostic accuracy of the immunoprofile system compared to the TNM staging system was conducted by receiver operating characteristic (ROC) analysis.

## Results

### Clinicopathological characteristics of the two cohorts

The clinical characteristics of two independent cohorts of ESCC patients are summarized in Table 1. In the primary cohort, 68 males and 27 females were enrolled with the median age at the diagnosis being 58 years. The validation cohort included 37 males and 18 females, with the median age at the diagnosis being 59 years. 91.6% (87/95) of patients in the primary cohort and 94.5% (52/55) of cases in the validation cohort received R0 esophagectomy, respectively. Together, 18.7% of tumors were Stage I, predominantly T1bN0 lesions. Most patients presented with local advanced stage (Stage II tumors made up 36.0%, Stage III tumors made up 36.7%). Stage IVA lesions disease comprised the remaining 8.6% of cases. All patients who underwent R0 resection entered the follow-up period directly after surgery, while those who had R1 resection received adjuvant fluoropyrimidine-based chemoradiation. For locoregional recurrence, concurrent fluoropyrimidine or taxane-based chemoradiation was recommended. Systemic chemotherapy was given to patients with metastatic diseases. Those patients with poor tolerance or poor performance score received single agent regimen and/or palliative supportive care. No one received immunotherapy in their treatment course.

**Table 1:** The baseline characteristics of the primary cohort and the validation cohort

	The primary cohort (*n* = 95) No. of patients (%)	The validation cohort (*n* = 55) No. of patients (%)	*P*-value
Sex			0.579
Male	68 (71.6)	37 (67.3)	
Female	27 (28.4)	18 (32.7)	
Age (years)			0.342
Median	58	59	
Range	38–81	37–78	
Esophagectomy			0.729
R0	87 (91.6)	52 (94.5)	
R1	8 (8.4)	3 (5.5)	
Grade			0.870
G1	9 (9.5)	4 (7.3)	
G2	69 (72.6)	40 (72.7)	
G3	17 (17.9)	11 (20.0)	
T Stage			0.340
T1a + T1b	14 (14.7)	7 (12.7)	
T2	30 (31.6)	18 (32.7)	
T3	33 (33.7)	25 (45.5)	
T4a	18 (19.0)	5 (9.1)	
N Stage			0.272
N0	64 (67.4)	29 (52.8)	
N1	18 (18.9)	13 (23.7)	
N2	10 (10.5)	11 (20.0)	
N3	3 (3.2)	2 (3.5)	
TNM Stage			0.656
IA + IB	20 (21.0)	8 (14.5)	
IIA + IIB	34 (35.8)	20 (36.4)	
IIIA+IIIB	32 (33.7)	23 (41.8)	
IVA	9 (9.5)	4 (7.3)	

### Description of TIL infiltration and checkpoint expression

#### TIL density and distribution

We detected the density and distribution of CD8+ T cells, CD4+ T cells, Foxp3+ T cells and CD33+ MDSC infiltrating the tumor site (Additional file 2). All specimens in the primary cohort presented with CD8+ and CD4+ T cells infiltration, 52.6% (50/95) of tumors had Foxp3+ T cells infiltration and 69.5% (66/95) of cases had CD33+ MDSC infiltration. TIL infiltration was most prominent in the tumor mesenchyme while only sparse infiltration was observed within the tumor parenchyma. No TIL infiltration was evident in areas of necrosis or in areas of keratin pearls.

#### Checkpoint expression

Overall, 31.6% (30/95) of tumors presented with membranous PD-L1 expression in tumor cells (TCs), whereas 30.5% (29/95) of tumors with PD-L1 expression in immune cells (ICs). 44.2% (42/95) of tumors had PD-1+ T cells infiltration. Significant correlation of PD-L1 expression in TCs with PD-1+ T cell infiltration was found (χ^2^ = 4.432, *P* = 0.035), indicating the activation of the inhibitory PD-1/PD-L1 pathway in ESCC cases. The expression of other inhibitory checkpoints including Tim-3 and LAG-3, and activating checkpoints including OX-40 and ICOS were also examined. Tim-3+ TILs or LAG-3+ TILs were found in 37.9% (36/95) or 16.8% (16/95) of tumors, respectively; whereas OX-40+ TILs or ICOS+ TILs were presented in 88.4% (84/95) or 91.6% (87/95) of cases, respectively.

#### IDO1 expression

IDO1, as an important immunosuppressive molecule, was found to be mainly located in the cell cytoplasm, and the expression of IDO1 was observed both in TCs and ICs (Additional file 2). 89.5% (85/95) of cases presented with IDO1 expression in ICs and 43.2% (41/95) of tumors with IDO1 expression in TCs, demonstrating IDO1 might play critical roles in the formation of immunosuppressive microenvironment in ESCC.

### Correlation of immunological parameters with survival

#### Correlation of TIL density with survival

**Fig. 1:**
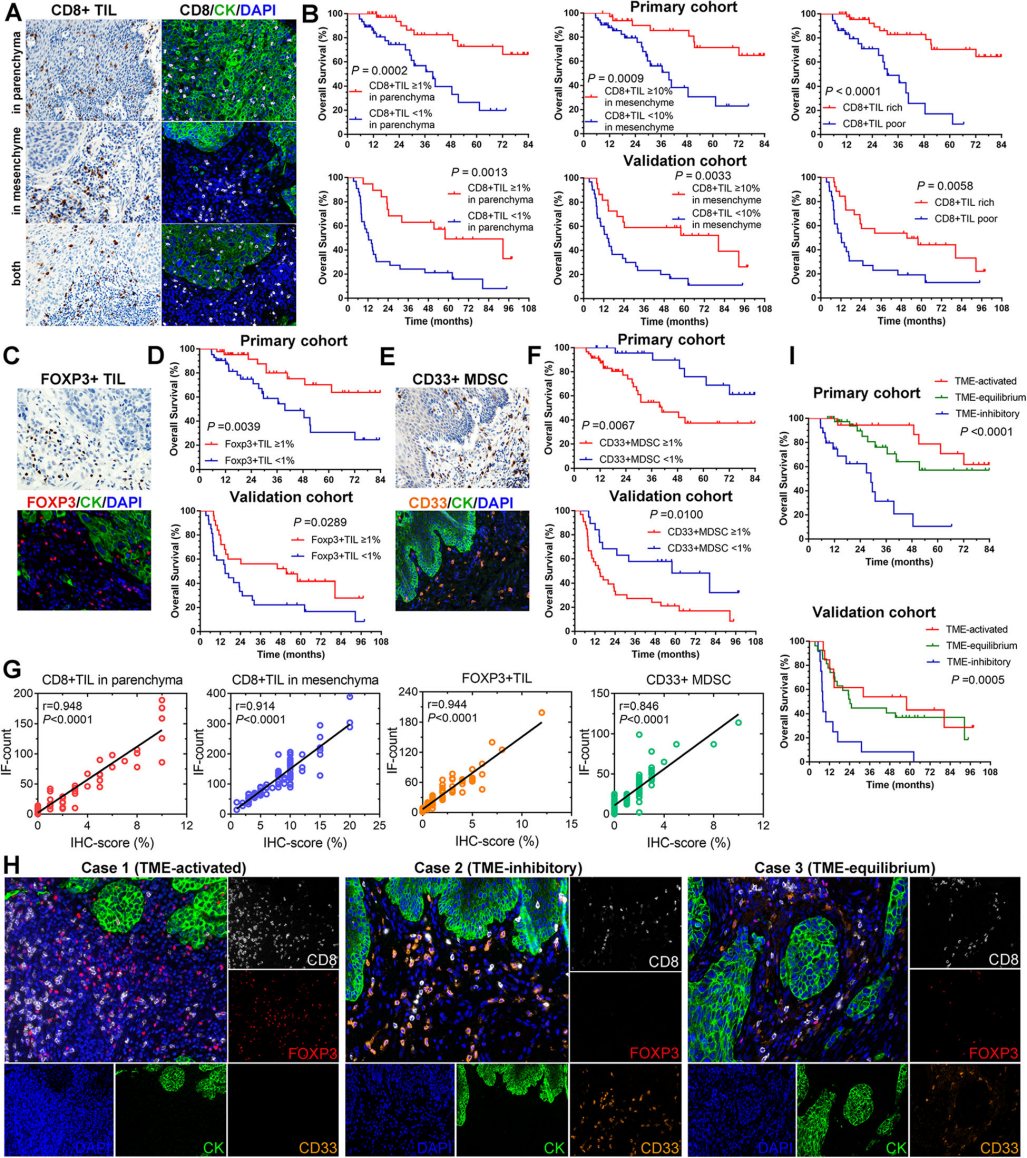
Correlation of immune cell infiltration and clinical outcome. **a** Immunohistochemistry (IHC) staining and multi-color immunofluorescence (IF) staining for CD8+ T cells infiltrating in parenchyma or mesenchyme or both (×200). **b** Overall survival of patients grouped by different CD8+ T cells infiltrating status. Less than 1% CD8+ T cells infiltrating in parenchyma and meanwhile less than 10% CD8+ T cells infiltrating in mesenchyme were defined as a poor infiltration; the others were regarded as a rich infiltration. **c** IHC staining and multi-color IF staining for FOXP3+ T cells (× 200). **d** Overall survival of patients grouped by different FOXP3+ T cells infiltrating status. **e** IHC staining and multi-color IF staining for CD33+ myeloid derived suppressor cells (MDSC) (× 200). **f** Overall survival of patients grouped by different CD33+ MDSC infiltrating status. **g** The correlation between the score of IHC staining and the automatic counting results of IF. **h** Representative IF images (× 200) of three different status of immune cell infiltration in the tumor microenvironment (TME). The more infiltration of immune effective cells was regarded as activated TME, the more infiltration of immune suppressive cells was defined as inhibitory TME, whereas the balanced infiltration of immune cells was considered as equilibrium. **i** Overall survival of patients grouped by different TME status. CD8 in white, FOXP3 in red, CD33 in orange and CK in green for multi-color IF staining. TIL, tumor-infiltrating lymphocytes

CD8+ and CD4+ T cells play a pivotal role in antitumor immunity. The densities of CD8+ and CD4+ immune infiltrates in the tumor parenchyma and tumor mesenchyme were assessed by IHC and multi-color IF analyses (Fig. 1a). The expression of CK was used to indicate tumor parenchyma in the multi-color IF analyses. We showed, for CD8 and CD4 markers, a statistically significant correlation between positive cell densities in each tumor region and patient outcome. Less than 1% CD8+ T cells infiltrating in parenchyma and meanwhile less than 10% CD8+ T cells (8% for CD4+ cells) infiltrating in mesenchyme were defined as a poor infiltration in total tumor area; the others were regarded as a rich infiltration. Longer OS was observed among patients with tumors containing a high density of such cells in parenchyma or mesenchyme or total tumor area than among patients whose tumors had low densities (Fig. 1b and Additional file 3).

The infiltration of Foxp3+ T cells and CD33+ MDSC into the tumor nest was rare, thus we calculated the densities of these cells in total tumor area. Patients with high density of Foxp3+ TILs showed an improved OS prognosis compared to patients with low density (Fig. 1c and d). Inversely, high density of CD33+ MDSC was associated with poor survival in ESCC patients compared to low MDSC density (Fig. 1e and f). Furthermore, the consistency between the results of IHC evaluation by experienced pathologists and the numbers of IF counting by software was analyzed, and the results showed a good agreement between the two approaches (Fig. 1g).

Different proportions of the above-mentioned TILs appear simultaneously in the TME, forming different status of TME and affecting clinical outcome (Fig. 1h and i). The amounts of CD8+ TIL, Foxp3+ TIL and CD33+ MDSC were counted automatically after the multi-color IF staining. Tumors with rich CD8+ TIL and positive Foxp3+ TIL infiltration in TME (TME-activated) tended to have favorable survival, whereas tumors with more immunosuppressive CD33+ MDSC (TME-inhibitory) was related to poorer clinical outcome. Nearly equal infiltration of immune effector cells and immunosuppressive cells was regarded as an equilibrium TME, in which tumors had superior survival than those in inhibitory TME. However, no significance was found between TME-equilibrium and TME-activated, indicating a more-defined scoring system to evaluate the status of TME.

#### Correlation of checkpoints expression with survival

**Fig. 2:**
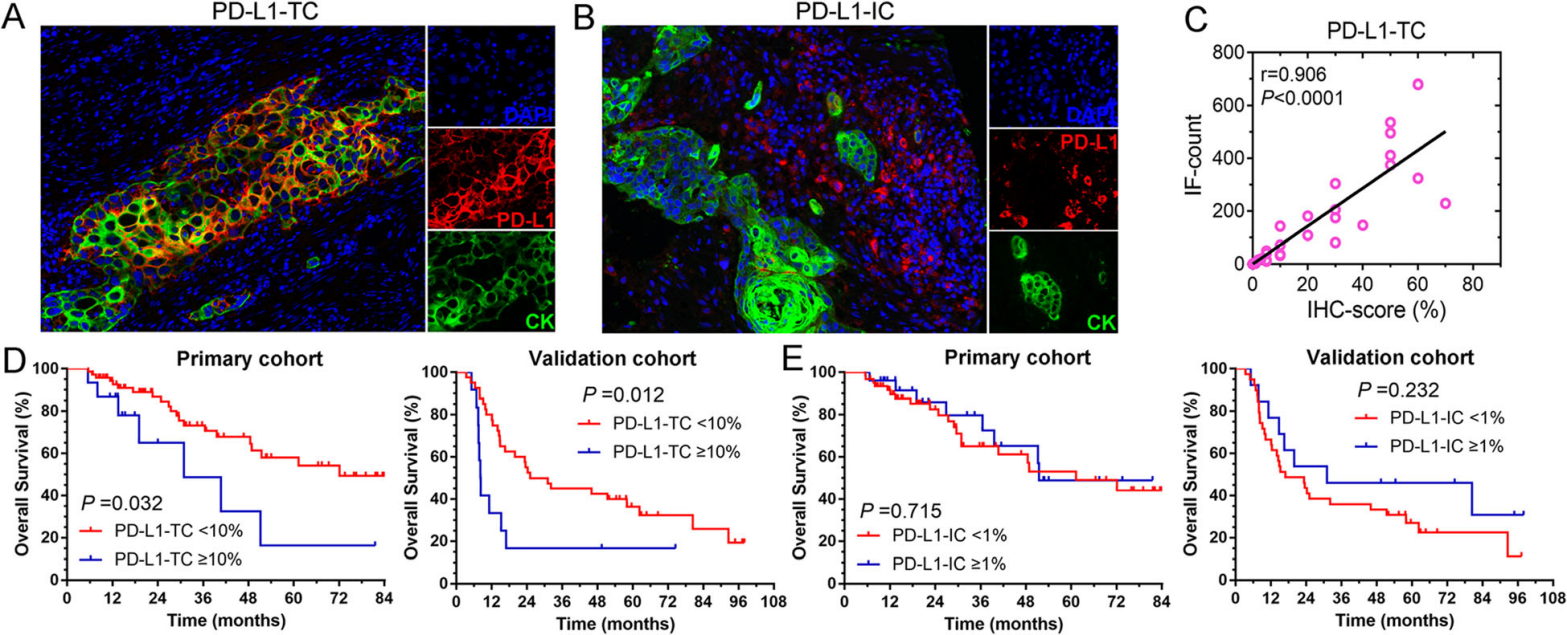
Correlation of checkpoint expression and clinical outcome. **a** and **b** Multi-color immunofluorescence (IF) staining for PD-L1 expression in tumor cells (TCs) or immune cells (ICs). **c** The correlation between the score of IHC staining and the automatic counting results of IF. **d** High density of PD-L1+ tumor cells predicted poor prognosis of esophageal squamous cell carcinoma. **e** Overall survival of patients grouped by different PD-L1 expression in ICs. TC, tumor cell; IC, immune cell

PD-L1 can be expressed by TCs and ICs (Fig. 2a and b), the prognostic value of PD-L1 expression by different cells in ESCC is unclear. IHC and IF were used to detect the PD-L1 expression, and the results of two methods accorded with each other (Fig. 2c). High positivity of PD-L1 expression (≥10%) in TCs was found to be associated with significantly lower survival in both cohorts (Fig. 2d), whereas the infiltration of PD-L1+ ICs was not associated with survival (Fig. 2e). There was no significant correlation between clinical outcome and the density of PD-1+ TILs (*P* = 0.214) either.

No information is by far available concerning the immune environment favoring the emergence of other checkpoints in ESCC. Tim3+ TILs tended to predict favorable prognosis of ESCC patients (*P* = 0.072), whereas no impact on survival prognosis was observed for LAG-3+ TILs (*P* = 0.185), OX-40+ TILs (*P* = 0.159) or ICOS+ TILs (*P* = 0.122) (Additional file 4).

#### Correlation of IDO1 expression with survival

The prognostic value of IDO1 expressed by different cells was analyzed. Neither the IDO1+ ICs (*P* = 0.273) nor IDO1+ TCs (*P* = 0.362) had statistically significant impact on survival (Additional file 4).

#### Correlation of TIL density and checkpoint expression

Based on the negative feedback mechanism of the immune system, immunosuppressive factors such as PD-L1 could be upregulated in response to immunologic effector cells infiltration. Thus, we analyzed the correlation of CD8+/CD4+ T cells infiltration and other TILs or checkpoints (Table 2). As expected, tumors were more likely to present with PD-L1+ TILs, Tim-3+ TILs and LAG-3+ TILs in a CD8-rich environment rather than a CD4-rich environment, indicating an adaptive immune status in ESCC. In addition, no association was observed between the infiltration of CD8+ T cells and clinicopathological parameters, whereas CD4-rich was correlated to better T stage and AJCC stage as shown by Additional file 5.

**Table 2:** Association between TILs and other immunological biomarkers in the primary cohort (*n* = 95)

Biomarker	CD8 rich (*n* = 56)	CD8 poor (*n* = 39)	*P* value	CD4 rich (*n* = 76)	CD4 poor (*n* = 19)	*P* value
	No. of patients	No. of patients		No. of patients	No. of patients	
Foxp3			**0.002**			**0.010**
≥ 1%	37	13		45	5	
< 1%	19	26		31	14	
CD33			0.682			0.119
≥ 1%	38	28		50	16	
< 1%	18	11		26	3	
PD-1			0.919			0.470
≥ 1%	25	17		35	7	
< 1%	31	22		41	12	
PD-L1-TC			0.555			0.581
≥ 1%	19	11		23	7	
< 1%	37	28		53	12	
PD-L1-TC			0.745			0.359
≥ 10%	10	8		13	5	
< 10%	46	31		63	14	
PD-L1-IC			**0.026**			0.316
≥ 1%	22	7		25	4	
< 1%	34	32		51	15	
Tim-3			**0.040**			0.245
≥ 1%	26	10		31	5	
< 1%	30	29		45	14	
LAG3			**0.047**			0.064
≥ 1%	13	3		16	0	
< 1%	43	36		60	19	
OX40			0.535			0.107
≥ 2%	42	27		58	11	
< 2%	14	12		18	8	
ICOS			0.200			0.354
≥ 6%	29	15		37	7	
< 6%	27	24		39	12	
IDO1-TC			0.441			0.679
≥ 1%	26	15		32	9	
< 1%	30	24		44	10	
IDO1-IC			0.616			0.172
≥ 6%	17	10		24	3	
< 6%	39	29		52	16	

*TILs* tumor infiltrating lymphocytes, *TC* tumor cell, *IC* immune cell

All entries in boldface are below 0.05

### Development of the nomogram-based immunoprofile

#### Independent prognostic factors of patients

Clinical characteristics and immune effectors of the primary cohort were included and subjected to univariable analysis. Among the clinical parameters, depth of tumor invasion (T stage), status of lymph node metastasis (N stage) and TNM stage were significantly associated with survival (Additional file 6). The risk factors with *P* value less than 0.1 in univariable analysis were selected into a Cox proportional hazards regression model to conduct multivariate analysis. Since the TNM stage contains T stage and N stage, we only put TNM stage into the multivariate analysis. At last, one tumor characteristic (TNM stage) and four immune variables (CD8, Foxp3, CD33, PD-L1-TC) had independent prognostic value for the OS of ESCC patients (Table 3).

**Table 3:** The univariate analyses and multivariate analyses for overall survival in the primary cohort

Prognostic Factor	Univariate analyses			Multivariate analyses			Score
	HR	95% CI	*P*	HR	95% CI	*P*	
Gender							
Male	1						
Female	1.222	0.555–2.703	0.621				
Age (years)							
< 60	1						
≥ 60	1.595	0.700–3.631	0.266				
History of smoking							
No	1						
Yes	1.717	0.728–4.049	0.217				
History of drinking							
No	1						
Yes	1.164	0.550–2.463	0.691				
AJCC TNM Stage							
I	0.039	0.007–0.212	**< 0.001**	0.056	0.007–0.429	**0.006**	**0**
II	0.103	0.025–0.416	**0.001**	0.061	0.011–0.343	**0.001**	**1**
III	0.181	0.047–0.690	**0.012**	0.088	0.020–0.391	**0.001**	**2**
IVA				1			**3**
CD8							
poor	1			1			**2**
rich	0.172	0.074–0.397	**< 0.001**	0.353	0.127–0.981	**0.046**	**0**
CD4							
poor	1			1			
rich	0.191	0.081–0.450	**< 0.001**	1.199	0.431–3.337	0.729	
Foxp3							
< 1%	1			1			**2**
≥ 1%	0.327	0.147–0.726	**0.006**	0.215	0.080–0.582	**0.002**	**0**
CD33							
< 1%	1			1			**0**
≥ 1%	3.348	1.334–8.404	**0.010**	2.956	1.026–8.517	**0.045**	**1.5**
PD-1							
< 1%	1						
≥ 1%	0.607	0.274–1.345	0.219				
PD-L1-TC							
< 10%	1			1			**0**
≥ 10%	2.498	1.052–5.931	**0.038**	3.131	1.099–8.921	**0.033**	**1.5**
PD-L1-IC							
< 1%	1						
≥ 1%	0.858	0.376–1.954	0.715				
Tim-3							
< 1%	1			1			
≥ 1%	0.478	0.210–1.088	**0.078** ^#^	0.405	0.148–1.103	0.077	
LAG3							
< 1%	1						
≥ 1%	0.391	0.093–1.652	0.201				
OX-40							
< 2%	1						
≥ 2%	0.577	0.266–1.253	0.164				
ICOS							
< 6%	1						
≥ 6%	0.539	0.243–1.194	0.128				
IDO1-TC							
< 1%	1						
≥ 1%	1.410	0.671–2.962	0.364				
IDO1-IC							
< 6%	1						
≥ 6%	1.515	0.718–3.198	0.276				

*HR* hazard ratio, *CI* confidence interval, *TC* tumor cell, *IC* immune cell, ^#^The risk factors with *P* value less than 0.1 in univariable analysis were selected into multivariate analysis

#### Development of nomogram-based immunoprofile and validation

**Fig. 3:**
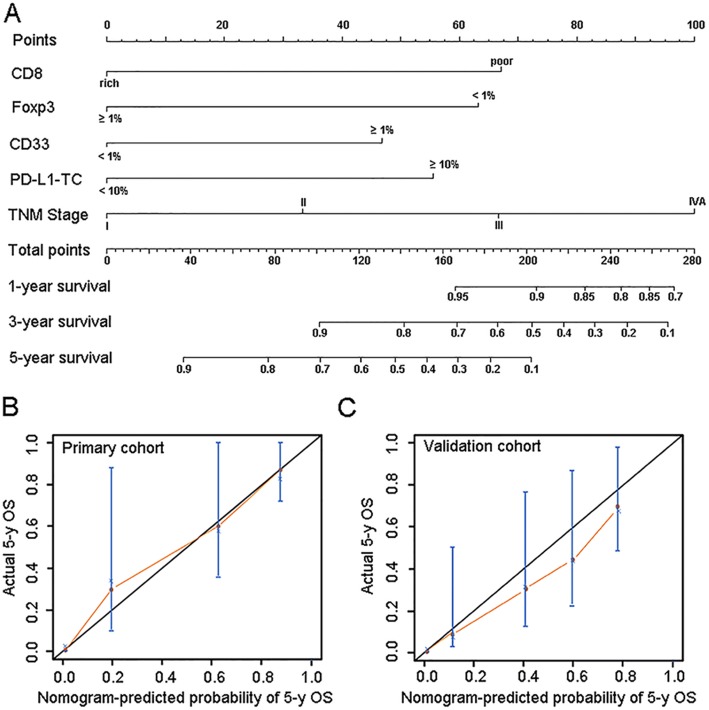
Development of the prognostic nomogram. **a** The nomogram for predicting overall survival in patients with ESCC after esophagectomy. To estimate the survival rate of an individual patient, the value of each factor is acquired on each variable axis, followed by a line drawn straightly upward to determine the points. The sum of these five numbers is located on the Total Points axis, then a line is drawn downward to the survival axes to determine the likelihood of survival. **b** and **c** The calibration curves for predicting patient overall survival (OS) in the primary cohort (**b**) and the validation cohort (**c**). The nomogram-predicted probability of survival is plotted on the x axis, and the actual survival is plotted on the y axis. TC, tumor cell

Although four immune variables had independent prognostic significance, their complicated interaction during the anti-tumor immune responses prevented any one of these factors from providing accurate predictions of survival in ESCC patients. Therefore, we need to develop a comprehensive immunoprofile for the prediction of survival. Based on the R software, nomogram for OS prediction were established using the five prognostic factors (Fig. 3a). The C-index for predicting OS was 0.844 (95% CI, 0.707–0.981) in the internal validation and 0.772 (95% CI, 0.617–0.927) in the external validation cohort, respectively, demonstrating that it is a model with favorable discriminative capability. The calibration curve for the probability of 5-year OS showed an optimal agreement between the actual observed survival and the prediction by the novel nomogram in the two cohorts (Fig. 3b and c), indicating the good predictive accuracy of the nomogram.

However, it is a little difficult for the wide clinical application of this prognostic nomogram due to its elaborate calculation process. We then modified this nomogram into a simple immunoprofile system based on the point of each factor in the nomogram. In the immunoprofile system, 0, 1, 2, 3 points correspond to Stage I, Stage II, Stage III, Stage IV, respectively. The points of CD8-poor and Foxp3 + TIL < 1% are near to that of Stage III in the nomogram, so 2 points are given to the two cases in the immunoprofile. The points of CD33+ MDSC ≥1% and PD-L1-TC ≥10% are between that of Stage II and that of Stage III in the nomogram, thus 1.5 points are given in the immunoprofile. Each patient was then assigned an immunoprofile index by summing the values of these variables. Patients with different immunoprofile indices showed different clinical outcome (Additional file 7).

#### Comparison of predictive accuracy between the immunoprofile and TNM stage system

**Fig. 4:**
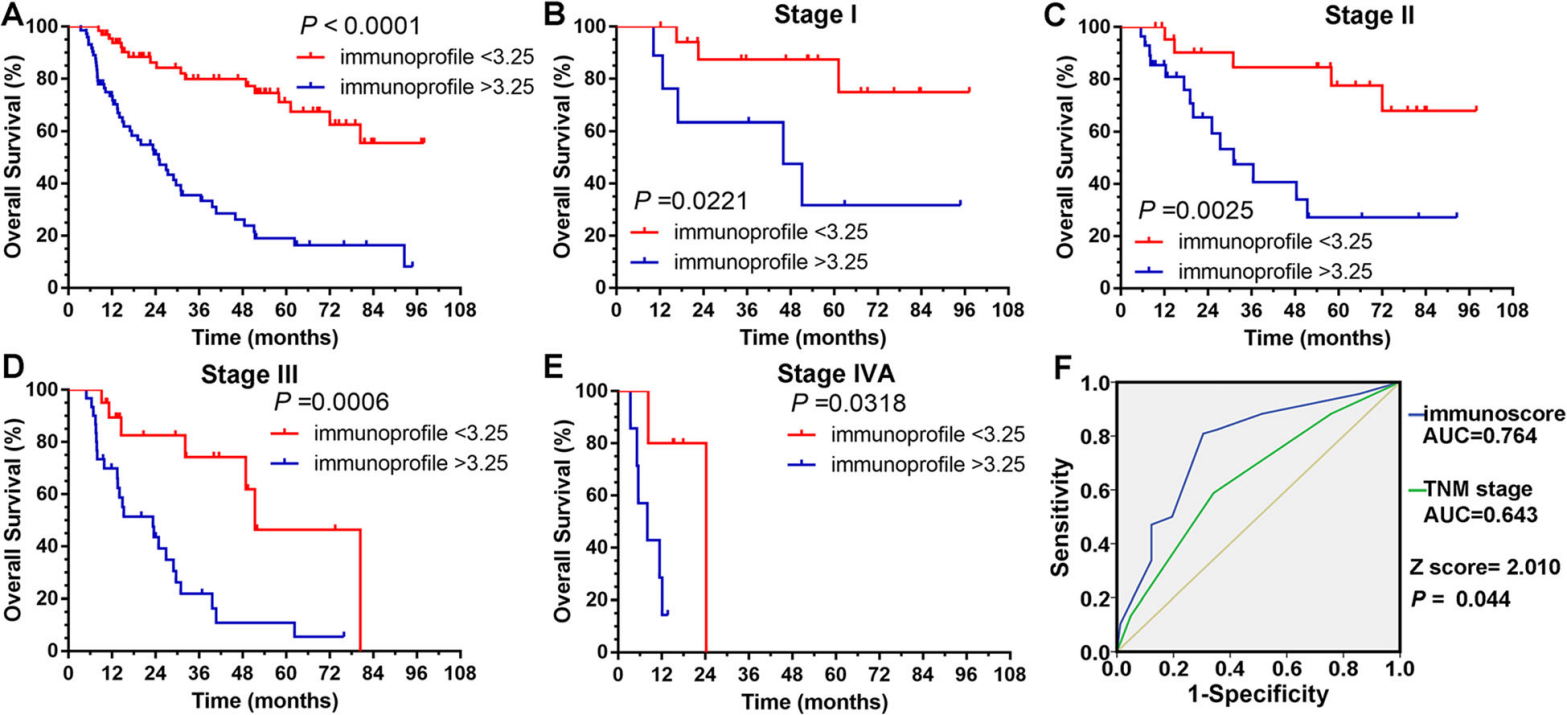
Survival curves based on the four-immune factor immunoprofile system. **a** The expression of PD-L1 in tumor cells and the infiltration of CD8+/Foxp3+/CD33+ cells have separated all patients into different risk subgroups using a value of 3.25. **b-e** Same-stage patients could be separated into different risk subgroups by the immunoprofile. **f** ROC analyses of 5-year survival rate based on the immunoprofile system and TNM stage for esophageal squamous cell carcinoma patients (*n* = 150). AUC, area under the curve

The discriminative value of the four immune variables in the immunoprofile system was further analyzed in the same-stage patients. Irrespective of the TNM stage, the four immune variables (expression of PD-L1 and infiltration of CD8+/Foxp3+ /CD33+ cells) have separated all patients into different risk subgroups using a value of 3.25 (Fig. 4a). 75.8% with score below 3.25 for the observation period were still alive, whereas 64.4% patients with a high score (above 3.25) died during the period. The immunoprofile system could split patients into different risk groups who were in the same stage (Fig. 4b-e), which may improve the prognostic accuracy of TNM staging.

To verify the accuracy of the immunoprofile compared to the TNM staging system, C-index was calculated and ROC analysis was performed. The C-indices for OS prediction were 0.802 and 0.702 by immunoprofile in the primary and validation cohorts, respectively; whereas the C-indices for OS prediction were 0.700 and 0.655 by TNM staging system in the primary and validation cohorts, respectively, all were inferior to the immunoprofile. Furthermore, our model yielded an area under the ROC curve (AUC) of 0.764 for prediction of mortality at 5 years, which was superior to TNM staging system with an AUC of 0.643 (Fig. 4f). These results suggested that the immunoprofile possesses an improved predictive accuracy for clinical outcomes in patients with ESCC compared to TNM stage.

## Discussion

Accumulating evidence has suggested that tumor progression is determined not only by its intrinsic characteristics, but also by extrinsic immunological factors in TME. For comprehensive estimation of immune parameters, the concept of “immune contexture” was proposed, which is defined by the type, density and location of immune cells within distinct tumor regions [27]. To improve the application of immune cell infiltration estimation in a clinical setting, the immunoscore concept was applied. The first established immunoscore only take the amount and location of CD3/CD8+ lymphocyte infiltration into account, other immune cells and critical factors such as immune checkpoints that could affect the interaction between tumor cells and immune cells were not included. This is the first time to analyze 11 different immunologic variables (the infiltration density of four immune cell types, the expression levels of six checkpoints as well as IDO1) in the TME at once and to construct an immune-related prognostic nomogram in patients with ESCC. To distinguish it from the previous algorithm of immunoscore, we defined our system as “immunoprofile”. Based on the excellent quantification capacity of nomogram, a simple and easily clinically used immunoprofile was established. Our immunoprofile model yielded an AUC of 0.764 for prediction of mortality at 5 years, which was superior to TNM stage (0.643) for patients with operable non-metastatic squamous cell carcinoma of the esophagus.

Our model utilizes four immune variables which are significantly associated with clinical outcomes for ESCC, including the infiltration of CD8+/Foxp3+/CD33+ cells and the expression of PD-L1 by tumor cells. As the most important immune effector, both the density and location of CD8+ TILs affect the anti-tumor immune response. The prognostic effect of increased density of CD8+ TILs was comprehensively reported in various cancers [28–30]. Compared to the independent prognostic impact of stromal CD8+ TILs in resected NSCLC [31], the infiltration of CD8+ TILs in parenchyma was more essential for ESCC. As shown in Additional file 3, tumors with positive CD8+ T cell infiltration in parenchyma whereas less infiltration in stroma have similar favorable survival with those with rich infiltration in both areas (the red curve vs. the green one), indicating that the prognosis will be better if there is evidence of CD8+ TIL in parenchyma regardless of the density in stroma. On the contrary, no impact on survival was observed for the infiltration of CD4+ T cells in parenchyma, resulting from the various CD4+ cell subtypes with distinct immune-helper or immunosuppressive roles, which is accordance with other studies on CD4+ T cells [30].

The prognostic value of Foxp3+ Tregs in cancer remains controversial. High infiltration of Foxp3+ Tregs possessed capacity to predict longer survival in our patients with ESCC. A meta-analysis [32] including 17 types of cancer showed that the prognostic effect of Foxp3+ Tregs varied greatly according to tumor site. High infiltration of Foxp3+ Tregs was significantly associated with shorter survival in the majority of solid tumors studied, such as renal, melanomas, and breast cancers; whereas Foxp3+ Tregs were associated with improved survival in colorectal, head and neck, and esophageal cancers, suggesting the organ local microenvironment may significantly influence the function of Foxp3+ Tregs. Together with the evidence of the positive effect of Foxp3+ Tregs on survival in esophageal adenocarcinoma [33], the immune microenvironment characteristics of the esophagus needs deep exploration to figure out which factors lead to the difference in the role of Foxp3 + Tregs.

MDSCs represent a heterogeneous population of cells with immunosuppressive properties and might confer to worse prognosis in cancer patients. Circulating CD33+ MDSCs were independently associated with decreased survival in various cancer types including esophageal cancer [34, 35]. Low levels of circulating CD33+ MDSCs before anti-CTLA-4 therapy correlated with an improved clinical response and long-term survival, indicating the predictive role of MDSC in checkpoint blockade [36]. However, rare studies focused on the effects of MDSC infiltration in situ, we analyzed this and found that the infiltration of CD33+ MDSC in situ was associated with increased risk of death in ESCC. At present, researchers concerned more about the role of CD8+ T cell infiltration in situ as a predictive biomarker for checkpoint blockade [37]. Our study might clue to the potential of CD33+ MDSC infiltration in situ as promising biomarker for checkpoint inhibitors, owing to the major immunosuppressive function of MDSC in the TME of ESCC we found.

As shown in Fig. 1h and i, patients with ESCC can be divided into three different subgroups based on the infiltration of immune effectors (CD8+/Foxp3+ TILs) and immunosuppressive cells (CD33+ MDSC) in the TME, indicating the critical role of TME in judging prognosis. The TME was before divided into four classifications according to the expression of PD-L1 in tumor cells and the presence of TILs [38], thus the expression of checkpoints was included into immunoscore studies. Previous studies reported the relationship between PD-L1+ tumors and poorer prognosis in ESCC [39, 40], however, they did not consider the expression of PD-L1 in TCs or ICs separately. This is the first time to investigate whether PD-L1 expressed by different cells has different prognostic values in ESCC. The expression of PD-L1 in TCs was regulated by two major mechanisms; the one is extrinsic immune-induced PD-L1 expression and the other is intrinsic oncogenic activation. Our study showed that the PD-L1 expression in TCs predicted unfavorable clinical outcome and was not associated with CD8+ T cell infiltration, indicating the upregulated PD-L1 expression in ESCC might be modulated mainly by intrinsic oncogenic activation. In contrast, more PD-L1+ ICs were found in the CD8-rich TME, which is in accordance with the feedback nature of immune system. In addition to PD-L1+ ICs, there existed more Tim-3+ ICs and LAG-3+ ICs in the CD8-rich TME, indicating that a pre-existing antitumor immune response might be tune off by these checkpoints and checkpoint blockade might be effective.

Based on four independently prognostic immune variables, our immunoprofile system demonstrated good levels of accuracy for survival prediction. However, there are some limitations. First, the sample size is relatively small and the performance of our model waits to be tested in larger cohorts. Second, the immune variables we used in this study were rather limited, with the development of cancer immunology research, sustained refinement of the immunoprofile is undoubtedly necessary, in which more and different prognostic factors might be included in the model. Furthermore, this study design excluded unresectable tumors or distant metastases, so results cannot be generalized to entire TNM staging system as M1 were excluded, and T4 population was extremely small.

## Conclusions

In this study, we built a nomogram-based immunoprofile system based on four immune variables. This comprehensive immunoprofile can improve the predictive accuracy of TNM staging system and is an important complement for the TNM system for operable ESCC patients. Future studies are needed to measure many other critical immune markers for inclusion in the system.

## Additional files

Additional file 1: **Table S1.** Primary antibodies used in IHC/IF. (PDF 81 kb)

Additional file 2: **Figure S1.** Representative IHC images of CD8, CD4, Foxp3, CD33, PD-1, PD-L1, TIM3, LAG3, OX-40, ICOS and IDO1 staining in esophageal squamous cell carcinoma samples (×200). TC, tumor cell; IC, immune cell. (PDF 416 kb)

Additional file 3: **Figure S2.** Survival curves of patients grouped by different CD8+ T cells infiltrating status (A) and different CD4+ T cells infiltrating status (B-E) in the primary cohort. TIL, tumor-infiltrating lymphocytes. (PDF 180 kb)

Additional file 4: **Figure S3.** Survival curves grouped by different Tim-3+ T cells infiltrating status (**A**), different LAG3+ T cells infiltrating status (B), different OX-40+ T cells infiltrating status (C), different ICOS+ T cells infiltrating status (D) and different IDO expression (E and F) in the primary cohort. TIL, tumor-infiltrating lymphocytes. IC, immune cell; TC, tumor cell. (PDF 175 kb)

Additional file 5: **Table S2.** Association between TILs and clinicopathological parameters in the primary cohort. (PDF 173 kb)

Additional file 6: **Figure S4.** Survival curves grouped by different T stages (A), N stages (B) and TNM stages (C) in all patients with ESCC (*n*=150). (PDF 162 kb)

Additional file 7: **Figure S5.** Survival curves grouped by immunoprofile in all patients with ESCC (*n*=150). (PDF 124 kb)
