# Supplementary material for: A nomogram-based immunoprofile predicts overall survival for previously untreated patients with esophageal squamous cell carcinoma after esophagectomy
Source: J Immunother Cancer. 2018 Oct 3;6:100. doi: 10.1186/s40425-018-0418-7 (PMC6171172; doi:10.1186/s40425-018-0418-7)
Supplement: Supplementary file 1 — Table S1. Primary antibodies used in IHC/IF. (PDF 81 kb) [file 40425_2018_418_MOESM1_ESM.pdf]

Supplementary Table S1. Primary antibodies used in IHC/IF

Species	Antigen	Source	Clone	Supplier	Dilution (IHC/IF)
Human	CD8	Rabbit	SP16	ZSBio	1:50/1:100
Human	CD4	Rabbit	EPR6855	Abcam	1:1200
Human	Foxp3	Mouse	236A/E7	Abcam	1:300/1:1000
Human	CD33	Rabbit	SP266	Abcam	1:500/1:500
Human	PD-1	Mouse	EH33	Cell Signaling Technology	1:200
Human	PD-L1	Rabbit	E1L3N	Cell Signaling Technology	1:200/1:1000
Human	Tim-3	Rabbit	D5D5R	Cell Signaling Technology	1:500
Human	LAG3	Rabbit	D2G4O	Cell Signaling Technology	1:200
Human	OX40	Rabbit	D1S6L	Cell Signaling Technology	1:100
Human	ICOS	Rabbit	D1K2T	Cell Signaling Technology	1:200
Human	IDO1	Rabbit	D5J4E	Cell Signaling Technology	1:800
Human	CK	Mouse	AE1/AE3	ZSBio	-/1:2000
