# Supplementary material for: A nomogram-based immunoprofile predicts overall survival for previously untreated patients with esophageal squamous cell carcinoma after esophagectomy
Source: J Immunother Cancer. 2018 Oct 3;6:100. doi: 10.1186/s40425-018-0418-7 (PMC6171172; doi:10.1186/s40425-018-0418-7)
Supplement: Supplementary file 2 — Figure S1. Representative IHC images of CD8, CD4, Foxp3, CD33, PD-1, PD-L1, TIM3, LAG3, OX-40, ICOS and IDO1 staining in esophageal squamous cell carcinoma samples (×200). TC, tumor cell; IC, immune cell. (PDF 416 kb) [file 40425_2018_418_MOESM2_ESM.pdf]

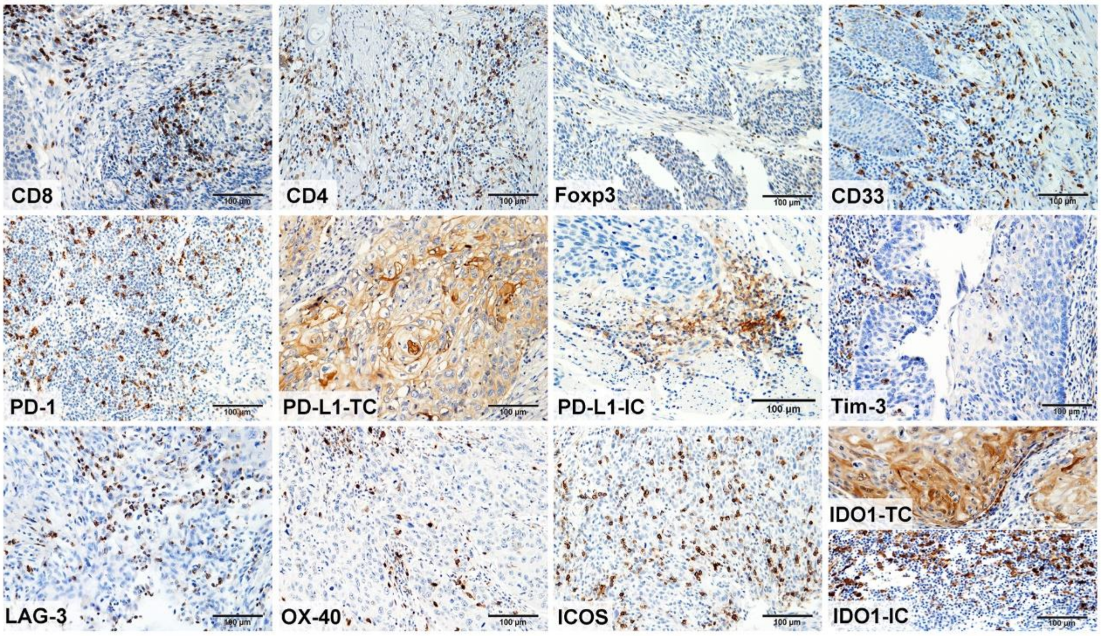

Supplementary Figure S1. Representative IHC images of CD8, CD4, Foxp3, CD33, PD-1, PD-L1, TIM3, LAG3, OX-40, ICOS and IDO1 staining in esophageal squamous cell carcinoma samples (×200). TC, tumor cell; IC, immune cell.
