# Supplementary material for: A nomogram-based immunoprofile predicts overall survival for previously untreated patients with esophageal squamous cell carcinoma after esophagectomy
Source: J Immunother Cancer. 2018 Oct 3;6:100. doi: 10.1186/s40425-018-0418-7 (PMC6171172; doi:10.1186/s40425-018-0418-7)
Supplement: Supplementary file 3 — Figure S2. Survival curves of patients grouped by different CD8+ T cells infiltrating status (A) and different CD4+ T cells infiltrating status (B-E) in the primary cohort. TIL, tumor-infiltrating lymphocytes. (PDF 180 kb) [file 40425_2018_418_MOESM3_ESM.pdf]

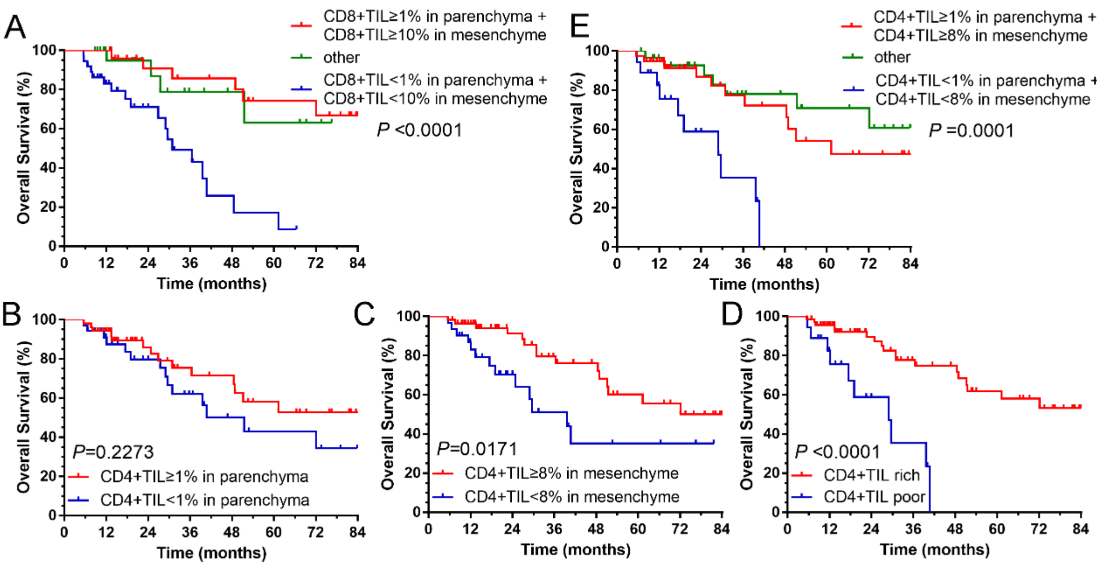

Supplementary Figure S2. Survival curves of patients grouped by different CD8+ T cells infiltrating status (**A**) and different CD4+ T cells infiltrating status (**B-E**) in the primary cohort. TIL, tumor-infiltrating lymphocytes.
