# Supplementary material for: A nomogram-based immunoprofile predicts overall survival for previously untreated patients with esophageal squamous cell carcinoma after esophagectomy
Source: J Immunother Cancer. 2018 Oct 3;6:100. doi: 10.1186/s40425-018-0418-7 (PMC6171172; doi:10.1186/s40425-018-0418-7)
Supplement: Supplementary file 4 — Figure S3. Survival curves grouped by different Tim-3+ T cells infiltrating status (A), different LAG3+ T cells infiltrating status (B), different OX-40+ T cells infiltrating status (C), different ICOS+ T cells infiltrating status (D) and different IDO expression (E and F) in the primary cohort. TIL, tumor-infiltrating lymphocytes. IC, immune cell; TC, tumor cell. (PDF 175 kb) [file 40425_2018_418_MOESM4_ESM.pdf]

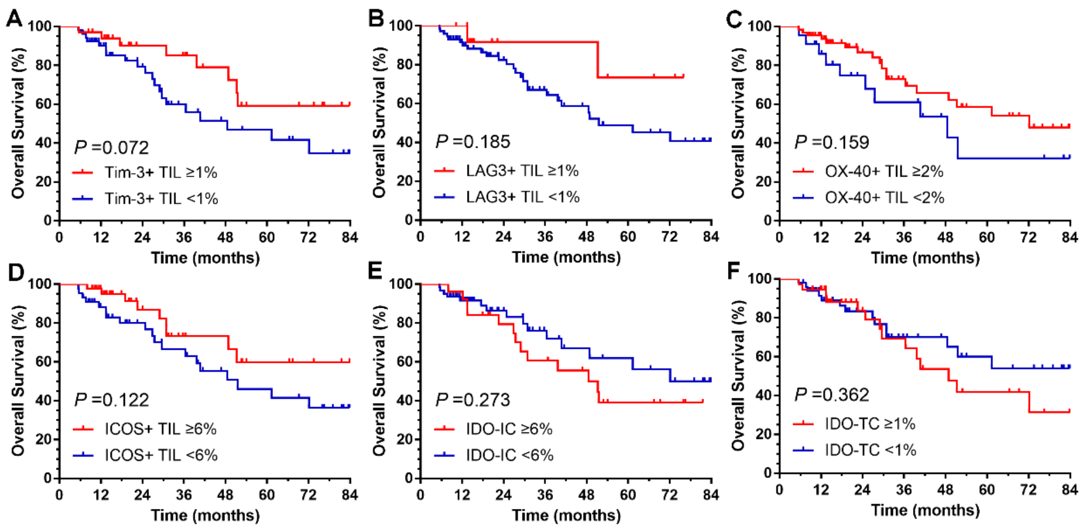


**Supplementary Figure S3.** Survival curves grouped by different Tim-3+ T cells infiltrating status (**A**), different LAG3+ T cells infiltrating status (**B**), different OX-40+ T cells infiltrating status (**C**), different ICOS+ T cells infiltrating status (**D**) and different IDO expression (**E and F**) in the primary cohort. TIL, tumor-infiltrating lymphocytes. IC, immune cell; TC, tumor cell.
