# Supplementary material for: A nomogram-based immunoprofile predicts overall survival for previously untreated patients with esophageal squamous cell carcinoma after esophagectomy
Source: J Immunother Cancer. 2018 Oct 3;6:100. doi: 10.1186/s40425-018-0418-7 (PMC6171172; doi:10.1186/s40425-018-0418-7)
Supplement: Supplementary file 5 — Table S2. Association between TILs and clinicopathological parameters in the primary cohort. (PDF 173 kb) [file 40425_2018_418_MOESM5_ESM.pdf]

Supplementary Table S2. Association between TILs and clinicopathological parameters in the primary cohort.

Characteristic	CD8 rich (n=56) No. of Patients	CD8 poor (n=39) No. of Patients	*P* value	CD4 rich (n=76) No. of Patients	CD4 poor (n=19) No. of Patients	*P* value
Sex			0.969			0.426
Male	40	28		53	15	
Female	16	11		23	4	
Age (years)			0.785			1.000
< 60	36	24		48	12	
≥ 60	20	15		28	7	
History of Smoking			0.785			0.595
Yes	36	24		47	13	
No	20	15		29	6	
History of Drinking			0.589			0.081
Yes	27	21		35	13	
No	29	18		41	6	
Location			0.280			0.855
Upper	4	4		6	2	
Middle	39	21		49	11	
Lower	13	14		21	6	
Length (cm)			0.766			0.631
median	3.9	4.0		3.9	4.1	
range	1.0-8.2	2.0-6.5		1.0-8.2	2.0-6.5	
Grade			0.157			0.060
G1	8	1		9	0	
G2	39	30		51	18	
G3	9	8		16	1	
T Stage			0.354			**0.046**
T1a+T1b	9	5		14	0	
T2	21	9		26	4	
T3	16	17		24	9	
T4a	10	8		12	6	
N Stage			0.808			0.194
N0	39	25		54	10	
N1	10	8		13	5	
N2	6	4		6	4	
N3	1	2		3	0	
TNM Stage			0.331			**0.014**
IA+IB	15	5		20	0	
IIA+IIB	18	16		27	7	
IIIA+IIIB	19	13		24	8	
IVA	4	5		5	4	

TILs, tumor infiltrating lymphocytes
