# Supplementary material for: A nomogram-based immunoprofile predicts overall survival for previously untreated patients with esophageal squamous cell carcinoma after esophagectomy
Source: J Immunother Cancer. 2018 Oct 3;6:100. doi: 10.1186/s40425-018-0418-7 (PMC6171172; doi:10.1186/s40425-018-0418-7)
Supplement: Supplementary file 6 — Figure S4. Survival curves grouped by different T stages (A), N stages (B) and TNM stages (C) in all patients with ESCC (n=150). (PDF 162 kb) [file 40425_2018_418_MOESM6_ESM.pdf]

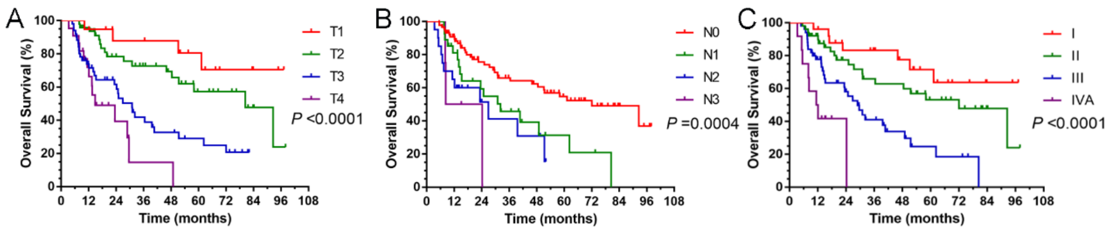

Supplementary Figure S4. Survival curves grouped by different T stages (**A**), N stages (**B**) and TNM stages (**C**) in all patients with ESCC (n=150).
