# Supplementary figures and images for: A nomogram-based immunoprofile predicts overall survival for previously untreated patients with esophageal squamous cell carcinoma after esophagectomy
Source: J Immunother Cancer. 2018 Oct 3;6:100. doi: 10.1186/s40425-018-0418-7 (PMC6171172; doi:10.1186/s40425-018-0418-7)

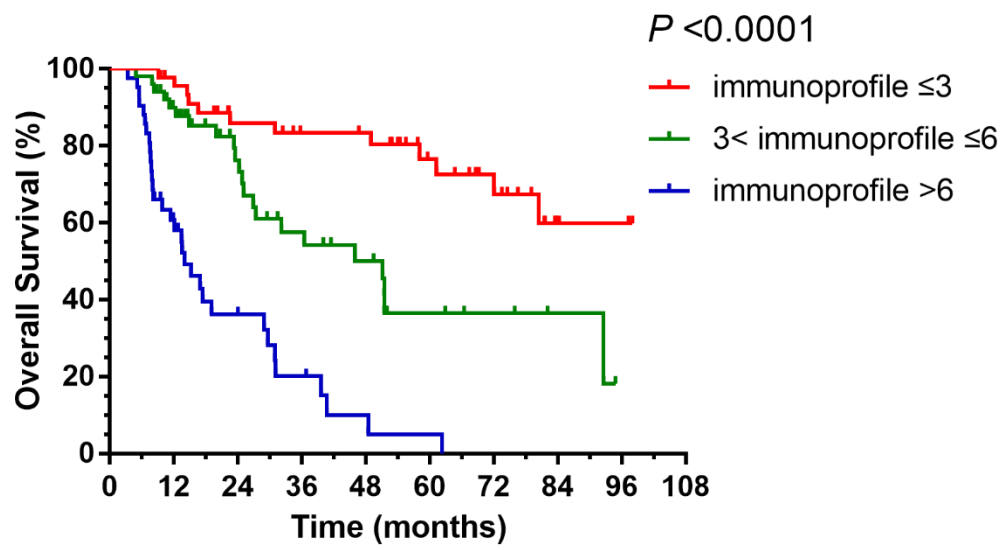

**Supplementary Figure S5.** Survival curves grouped by immunoprofile in all patients with ESCC (n=150).

Supplement: Supplementary file 7 — Figure S5. Survival curves grouped by immunoprofile in all patients with ESCC (n=150). (PDF 124 kb) [file 40425_2018_418_MOESM7_ESM.pdf]
